# Expression and function of *Caenorhabditis elegans* UNCP-18, a paralog of the SM protein UNC-18

**DOI:** 10.1093/genetics/iyad180

**Published:** 2023-10-05

**Authors:** Marion Boeglin, Eduardo Leyva-Díaz, Oliver Hobert

**Affiliations:** Department of Biological Sciences, Columbia University, Howard Hughes Medical Institute, NewYork, NY 10027, USA; Department of Development and Stem Cells, IGBMC, CNRS UMR 7104/INSERM U1258, Université de Strasbourg, Strasbourg 67081, France; Department of Biological Sciences, Columbia University, Howard Hughes Medical Institute, NewYork, NY 10027, USA; Department of Biological Sciences, Columbia University, Howard Hughes Medical Institute, NewYork, NY 10027, USA

**Keywords:** *C. elegans*, SM protein, secretory pathway, nervous system, behavior

## Abstract

Sec1/Munc18 (SM) proteins are important regulators of SNARE complex assembly during exocytosis throughout all major animal tissue types. However, expression of a founding member of the SM family, UNC-18, is mostly restricted to the nervous system of the nematode *Caenorhabditis elegans*, where it is important for synaptic transmission. Moreover, *unc-18* null mutants do not display the lethality phenotype associated with (a) loss of all *Drosophila* and mouse orthologs of *unc-18* and (b) with complete elimination of synaptic transmission in *C. elegans*. We investigated whether a previously uncharacterized *unc-18* paralog, which we named *uncp-18*, may be able to explain the restricted expression and limited phenotypes of *unc-18* null mutants. A reporter allele shows ubiquitous expression of *uncp-18*. Analysis of *uncp-18* null mutants, *unc-18* and *uncp-18* double null mutants, as well as overexpression of *uncp-18* in an *unc-18* null mutant background, shows that these 2 genes can functionally compensate for one another and are redundantly required for embryonic viability. Our results indicate that the synaptic transmission defects of *unc-18* null mutants cannot necessarily be interpreted as constituting a null phenotype for SM protein function at the synapse.

## Introduction

**Fig. 1.**
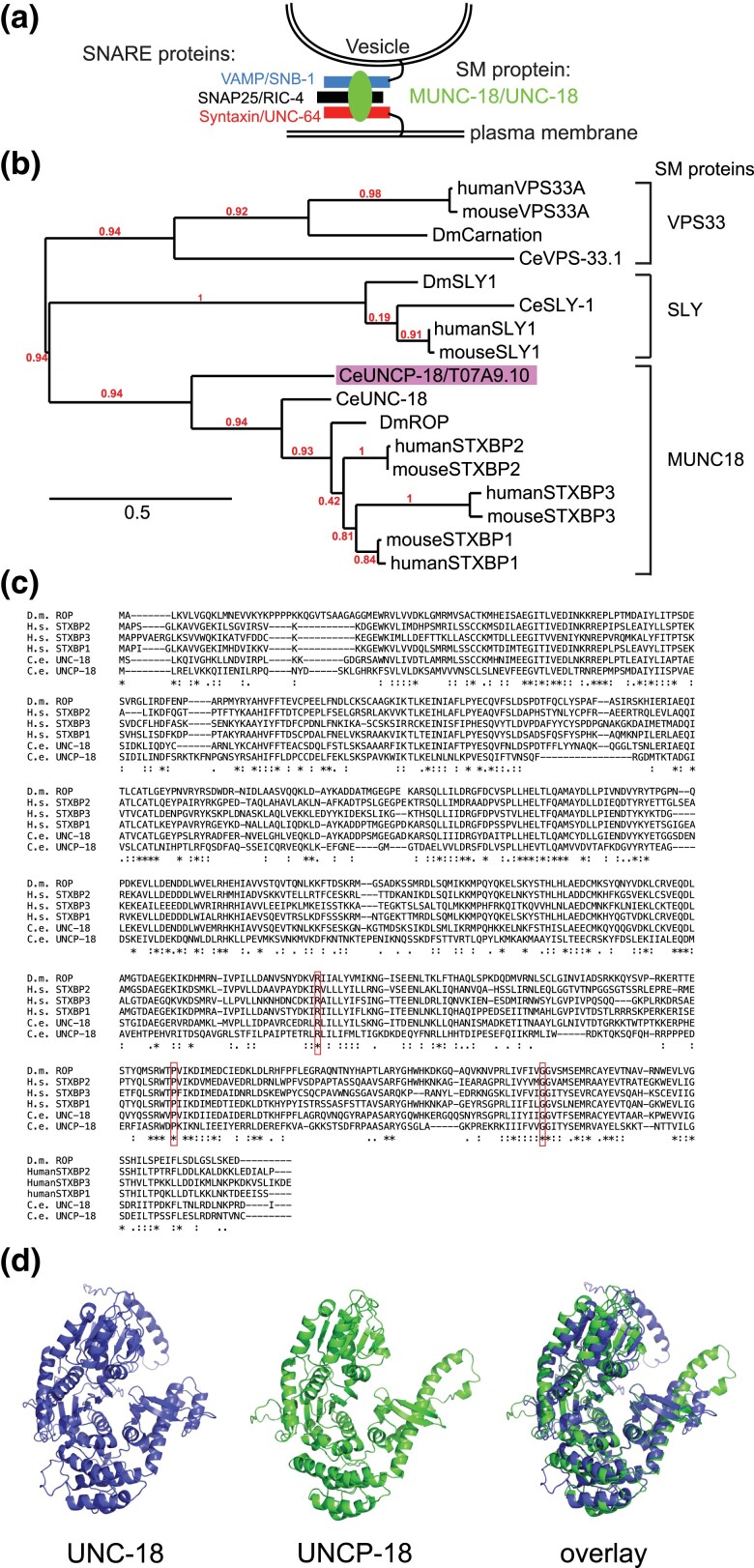
UNCP-18 is a paralog of UNC-18. a) Simplified representation of a SNARE complex. b) Phylogenetic tree built at phylogeny.fr (Dereeper et al. 2008). c) ClustalW alignment generated with T-Coffee. The boxed amino acids are mutated in human neurological disease and required for STXB1 and UNC-18 protein function and stability (Guiberson et al. 2018). Several of them are also conserved in other SM family members, indicating their importance for general SM protein function. d) AlphaFold structure prediction of UNCP-18 and UNC-18 (Jumper *et al.* 2021).

SNARE (*s*oluble *N*-ethylmaleimide-sensitive fusion protein *a*ttachment protein *re*ceptors) protein complexes are mediators of membrane fusion events and are employed in several essential cellular processes, including exocytosis in secretory cells (Han et al. 2017). Sec1/Munc18 (SM) proteins are involved in the assembly of SNARE complexes in a number of different contexts, including at synaptic vesicle release sites in the nervous system (Aalto et al. 1992; Sudhof and Rothman 2009; Zhang and Hughson 2021) (Fig. 1a). The nematode *Caenorhabditis elegans* contains several cell types that are secretory including neurons, intestinal cells, and several types of gland cells located in different parts of the body (Hall and Altun 2007). However, the UNC-18 protein, a founding member of the SM family (Brenner 1974; Aalto et al. 1992; Gengyo-Ando et al. 1993), is expressed only in the nervous system, the intestine, and the male gonad, but not in other secretory cells, such as the excretory, pharyngeal, or rectal gland cells (Gengyo-Ando et al. 1993; Schindelman et al. 2006; Stefanakis et al. 2015), raising the question whether another SM protein may assist in SNARE assembly in those cell types.

Within the nervous system, *unc-18* has been shown to be important for synaptic transmission (Hosono et al. 1992; Sassa et al. 1999; Weimer et al. 2003; McEwen and Kaplan 2008; Gracheva et al. 2010; Park et al. 2017). Nevertheless, the reported *unc-18* null mutant phenotype is less severe than the null mutant phenotype of other proteins involved in the synaptic vesicle cycle. While *unc-18* null mutant animals are severely impacted in locomotion (Sassa et al. 1999), they are viable, which contrasts with the phenotype of other SNARE complex components, such as v-SNARE synaptobrevin (*snb-1*) or the t-SNARE syntaxin (*unc-64)* mutants, both of which die as paralyzed first stage larvae (Nonet et al. 1998; Saifee et al. 1998). Similar lethality phenotypes are observed in animals that lack the synaptic vesicle regulator *unc-13* or are devoid of the synaptic vesicle cargo acetylcholine (Alfonso et al. 1993; Kohn et al. 2000). Genetic removal of fly and mouse *unc-18* homologs also results in animal lethality (Harrison et al. 1994; Verhage et al. 2000; Kanda et al. 2005; Kim et al. 2012) and phenocopies the loss of other synaptic vesicle cycle proteins, like Munc13 (He et al. 2017). Electrophysiological recording also indicate that neurotransmission is severely reduced, but not eliminated in *unc-18* mutant animals (Weimer et al. 2003).

One potential explanation for the restricted *unc-18* expression and limited severity of the null mutant phenotype compared to other synaptic vesicle regulators is the presence of a paralog of *unc-18* in the *C. elegans* genome, the previously uncharacterized T07A9.10 gene. We explore here the expression and function of this gene, as well as its genetic interactions with *unc-18*.

## Material and methods

### Strains

*uncp-18* deletion and reporter alleles *syb6377* and *syb6123*, respectively, were generated by Sunybiotech, using CRISPR/Cas9-based genome engineering. We generated the *unc-18(ot1432)* reporter allele using a newly described CRISPR/Cas9 engineering protocol (Eroglu et al. 2023). *uncp-18* was overexpressed in an *unc-18(e81)* by injecting fosmid WRM0635cH12 at 15 ng/µL final concentration with *inx6p18::gfp* as coinjection marker (Bhattacharya and Hobert 2019). Two resulting extrachromosomal arrays, *otEx8105* and *otEx8106*, were subjected to behavioral analysis.

### Locomotory behavior

Locomotory assays were performed using the WormLab automated multiworm tracking system (MBF Bioscience) (Roussel et al. 2014) at room temperature. In brief, 5 young adult animals were transferred into NGM and video-recorded for 5 min. Multiple features of their locomotory behavior were then analyzed using WormLab software. WormLab data were exported to Prism (GraphPad), and statistical significance between each group was calculated using Kruskal–Wallis test followed by Dunn's multiple comparisons.

### Aldicarb assays

Aldicarb assays were performed as previously described (Mahoney et al. 2006). Briefly, 25 young adult animals (24 h after L4 stage, blinded for genotype) were picked onto freshly seeded NGM plates containing 1 mM aldicarb (Chem Service). Worms were assayed for paralysis every 30 min by prodding with a platinum wire. A worm was considered paralyzed if it did not respond to prodding to the head and tail 3 times each at a given time point. Strains were grown and assayed at room temperature. Statistical significance between each group was calculated in Prism (GraphPad) using 2-way ANOVA followed by Tukey's multiple comparisons test.

### Brood size, hatching, and embryonic viability

A total of 20 late L4 hermaphrodite from an unstarved plate were picked to individual fresh plate and were incubated for 2 days at 20°C. These plates were then scored, and all the hatched larvae were removed until the individual worms were stop laying progeny. The same protocol was used to determine the hatching percentage; the total number of larvae were compared to the number unhatched eggs.

To score embryonic viability of *unc-18; uncp-18* double mutants, 4 individual youn adult stage *unc-18; uncp-18/+* hermaphrodites were picked to individual fresh plate. After a few hours at 20°C, about 16 fresh eggs were isolated from each plate and followed during the next days to determine whether they hatched into viable larvae.

### Microscopy

Worms were anesthetized using 100 mM of sodium azide and mounted on 5% agarose on glass slides. All images were acquired using a Zeiss confocal microscope (LSM 980). Image reconstructions were performed using Zen software tools. Maximum intensity projections of representative images were shown.

## Results and discussion

### T07A9.10/UNCP-18 is a sequence paralog of UNC-18

Reciprocal BLAST searches of UNC-18 protein sequences show close sequence similarity with a previously uncharacterized and unnamed gene, T07A9.10. Protein sequence comparison of T07A9.10 and other members of the SM family of proteins reveals that T07A9.10 clusters together with UNC-18, the *Drosophila*  UNC-18 homolog *Rop*, and their vertebrate orthologs STXBP1 (Munc18-1), STXBP2 (Munc18-2), and STXBP3 (Munc18-3)(Fig. 1, b and c). SM proteins from the SLY and VPS33 family, each involved in different intracellular trafficking and membrane fusion processes (Koumandou et al. 2007), cluster in separate branches (Fig. 1b). Phylogenetic sequence analysis also indicates that the duplication that generated UNC-18 and T07A9.10 occurred independently of the duplications that generated the 3 Munc18 protein paralogs (Fig. 1b).

De novo mutations in the *unc-18* ortholog STXBP1 are among the most frequent causes of epilepsy and encephalopathy (Stamberger et al. 2016). The effect of 3 of these disease-causing mutations in human *STXB1* (R406H, P480L, and G544D) on protein stability and function have been investigated in detail (Guiberson et al. 2018). These 3 amino acids are conserved in *C. elegans unc-18* where they are required for *unc-18* function (Guiberson et al. 2018). These amino acids are conserved in T07A9.10 as well (Fig. 1c). The similarity of the UNC-18 and T07A9.10 proteins can also be visualized by structural predictions using AlphaFold (Jumper et al. 2021). An overlay of the predicted structures shows that all major secondary structural elements and their overall arrangement into ternary structures are similar (Fig. 1d).

Taken together, these lines of evidence indicate that UNC-18 and T07A9.10 are paralogous genes, generated by gene duplication at the base of nematode evolution. We henceforth named the T07A9.10 gene *uncp-18* for “*unc-18*  p aralog.” Reciprocal BLAST searches show that other nematode species, within and outside the *Caenorhabditis* genus, also usually code for 2 separate *unc-18* paralogs.

### Expression pattern of UNCP-18

**Fig. 2.**
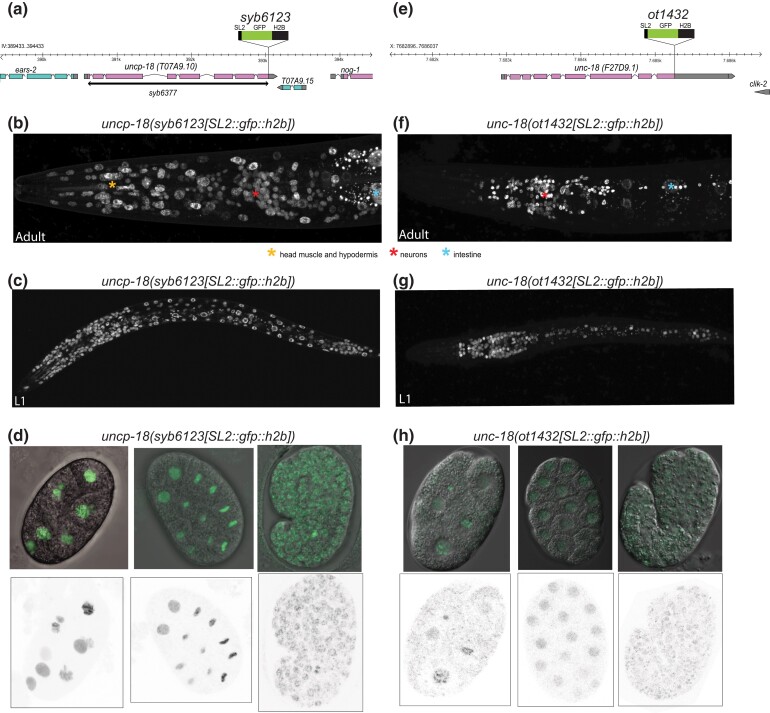
uncp-18  *and unc-18* locus and their expression patterns. a) *uncp-18* locus and CRISPR/Cas9-engineered null allele (*syb6377*) and reporter allele (*syb6123*). b–d) *uncp-18(syb6123[uncp-18::SL2::gfp::h2b])* animals at different developmental stages (b, adult head; c, first larval stage animal; and d, different embryonic stages). e) *unc-18* locus and CRISPR/Cas9-engineered reporter allele (*ot1432*). f–h) *unc-18(ot1432[unc-18::SL2::gfp::h2b])* animals at different developmental stages (f, adult head; g, first larval stage animal; and h, different embryonic stages).

We examined the expression of the *uncp-18* locus by inserting an SL2::GFP::H2B reporter cassette at the 3′ end of the *uncp-18* coding sequence through CRISPR/Cas9-assisted genome engineering (Fig. 2a). Through its polycistronic nature, this reporter cassette produces GFP in all cells in which *uncp-18* is expressed. To facilitate the identification of expressing cells, the GFP protein is targeted to the nucleus via the histone H2B moiety. We find that *uncp-18(syb6123[uncp-18::SL2::gfp::h2b])* animals show GFP expression in an apparently ubiquitous pattern in all cell types (Fig. 2b). Expression commences in very early stage embryos and the gene continues to be ubiquitously expressed throughout all stages of embryonic and larval development (Fig. 2, b, c, and d).

To compare the expression pattern of *uncp-18* with *unc-18*, we genome-engineered an *unc-18* reporter allele, *ot1432*, using the same SL2::GFP::H2B reporter cassette (Fig. 2e). We found *unc-18(ot1432[unc-18::SL2::gfp::h2b])* animals show GFP expression throughout the nervous and the intestine, as previously reported with a fosmid-based reporter (Fig. 2f and g) (Stefanakis et al. 2015). In addition, we observed previously unappreciated, very low level of expression of *unc-18* throughout all cells of the developing embryo (Fig. 2h).

### Null phenotype of *uncp-18*

**Fig. 3.**
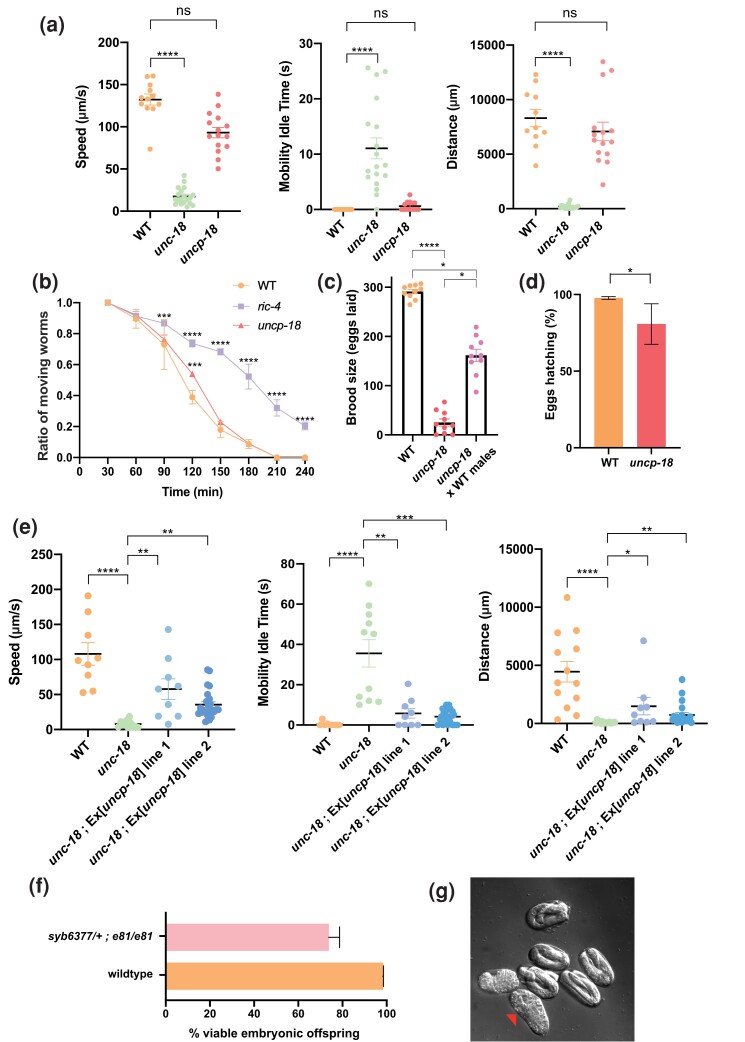
Analysis of an *uncp-18* null allele and genetic interactions with *unc-18.* a) Quantification of locomotory behavior using the WormLab automated multiworm tracking system (Roussel et al. 2014) reveals reveal no significant locomotory defects of *uncp-18(syb6377)* mutant animals. Each dot represents the indicated feature value for a single animal with the mean ± SEM indicated. These data have been collected on 2 different days and were then combined. For comparisons, the Kruskal–Wallis test followed by Dunn's multiple comparisons was used. ****P < 0.0001. *n* ≥ 10 for all genotypes. b) Aldicarb assays reveal no synaptic transmission defects in *uncp-18(syb6377)* mutant animals. Tests with aldicarb resistant *ric-4(md1088)* were done as control. Two-way ANOVA followed by Tukey's multiple comparisons test was done. ***P < 0.001, ****P < 0.0001. *n* = 3 independent experiments (25 animals per independent experiment). c) Reduced brood size of *uncp-18(syb6377)* mutant compared to P0 from the *uncp-18(syb6377)* with WT males cross and WT animals. The progeny of at least 20 hermaphrodites was scored at 20°C for each genotype until the worms stop laying progeny, starting with early adulthood. Error bars indicate the standard deviation across 2 separate individual replicates. Stasticial significance was calculated using the Kruskal-Wallis test *P < 0.05, ****P < 0.0001. d) *uncp-18(syb6377)* mutant animals produce unfertilized eggs, assessed by quantifying the percentage of eggs, laid by 10 single worms, that hatched to produced viable progeny. Statistics were calculated using Student's t-test. *P < 0.05 e) Overexpression of *uncp-18* partially rescues the locomotory defects of *unc-18* null mutants, measured with the WormLab automated multiworm tracking system. Each dot represents the indicated feature value for a single animal with the mean ± SEM indicated. The *n* corresponds to the combination of results collected on 2 different day, explaining the variability of the locomotory data of panel a. For comparisons, the Kruskal–Wallis test followed by Dunn's multiple comparisons was used. **P < 0.01, ***P < 0.001, ****P < 0.0001. *n* ≥ 10 for all genotypes. f, g) *unc-18(e81); uncp-18(syb6377)* double null mutants are embryonic lethal. Analysis of the viability of the offspring of 4 single *unc-18(e81); uncp-18(syb6377)/+* mothers shows that about ¼ of the offspring are inviable (f). g) A representative image of an arrested, disorganized animal (red arrowhead), seen alongside viable progeny at various stages of embryonic development. See also Supplementary Movie 1. Note that unlike *uncp-18* null mutants, which do not have any maternal contribution of *uncp-18* (hence resulting in apparently partially penetrant fecundity effects), the progeny of *unc-18(e81); uncp-18(syb6377)/+* mothers do have wild-type *uncp-18* maternal gene dosage.

To assess the function of *uncp-18*, we used CRISPR/Cas9 genome engineering to delete the entire *uncp-18* coding sequence, from the predicted start codon to the predicted stop codon (Fig. 2a). Animals carrying this null allele, *syb6377*, are fully viable. This contrasts the lethality observed upon genetic ablation of any of the broadly expressed vertebrate Munc18 homologs, Munc18-2 and Munc18-3 (Kanda et al. 2005; Kim et al. 2012) or the sole *Drosophila* homolog of *unc-18*, Rop (Harrison et al. 1994). *uncp-18* null mutant animals also display none of the obvious locomotory phenotypes often associated with defects in synaptic transmission in *C. elegans*, such as those observed in *unc-18* mutants (Fig. 3a). Several proteins involved in synaptic transmission only display phenotypes in response to AChE-inhibitor aldicarb (Miller et al. 1996), but *uncp-18* null mutants show an aldicarb response profile that is not obviously different from wild-type animals (Fig. 3b).

However, *uncp-18* mutant animals produce much fewer eggs compared to wild-type control animals (Fig. 3c). A fraction of the eggs produced by *uncp-18* mutants animals have the typical, dark appearance of unfertilized oocytes (Fig. 3d). We crossed wild-type males with *uncp-18* mutants and found that brood size defects were partially restored, even though not to a wild-type level (Fig. 3c). These results are consistent with gametogenesis defects of *uncp-18* mutants. We note that several secretory pathway mutants display membrane biogenesis defects during oogenesis (Hanna et al. 2013).

### Genetic interactions of *uncp-18* and *unc-18*

We next asked whether the locomotory defects of *unc-18* null mutant animals can be rescued by *uncp-18* overexpression. To this end, we provided extra copies of *uncp-18* through an extrachromosomal array that contains the *uncp-18-*containing fosmid WRM0635cH12. We found that in such transgenic animals the *unc-18* locomotory defects are partially rescued (Fig. 3e). Since *unc-18* and *uncp-18* are normally coexpressed in the nervous system, we interpret this finding to mean that either (a) the *unc-18* null phenotype (locomotory defects and synaptic transmission defects) is the result of lowering of the SM protein dosage (UNC-18 + UNCP-18), rather than a complete loss of SM protein function in the nervous system, or, (b), *unc-18* and *uncp-18* may have different biochemical functions at the synapse, but overexpression of UNCP-18 is able to substitute for the biochemical activity that UNC-18 normally fulfills.

To further investigate the possibility of redundant *unc-18/uncp-18* function, we generated *unc-18(e81); uncp-18(syb6377)* double null mutant animals. Our initial expectation was that such animals would arrest development at the first larval stage, like other synaptic transmission mutants (Nonet et al. 1998; Saifee et al. 1998). However, while animals that are homozygous for *unc-18(e81)* and heterozygous for *uncp-18(syb6377)* are fully viable, we were unable to identify double homozygous viable larval or adult offspring. We carefully followed the progeny of individual *unc-18(e81); uncp-18(syb6377)/+* animals and found that these animals produce about 25% inviable offspring (Fig. 3f), indicating fully penetrant embryonic lethality of *unc-18; uncp-18* double null mutant embryos. The morphology of inviable embryos appears highly disorganized (Fig. 3g). We video-recorded the offspring of a heterozygous animal and observed embryonic developmental arrest before any signs of overt morphogenesis (Supplementary Movie 1). We have not further investigated the cause of embryonic arrest but note that SNARE complexes are required for several different steps of embryogenesis, ranging from egg-shell secretion to cytokinesis (Jantsch-Plunger and Glotzer 1999; Sato et al. 2008; Kang et al. 2011). Other SNARE complex-dependent secretory processes essential for embryonic development may remain to be identified.

We conclude that UNC-18 and UNCP-18 operate synergistically during embryonic development. Given this embryonic synergy, it appears conceivable that UNC-18 and UNCP-18 may also have redundant functions at the synapse and, therefore, that the role of SM proteins at the *C. elegans* synapse was previously underappreciated.

## Supplementary Material

iyad180_Supplementary_Data

## Data Availability

Any additional information required to analyze the data reported in this paper is available from the lead contact upon request. Further information and requests for resources and reagents should be directed to and will be fulfilled by the Lead Contact, Oliver Hobert (or38@columbia.edu). All newly generated strains will be available at the Caenorhabditis Genetics Center (CGC). Supplemental material available at GENETICS online.
