# Supplementary material for: Expression and function of Caenorhabditis elegans UNCP-18, a paralog of the SM protein UNC-18
Source: Genetics. 2023 Oct 5;225(4):iyad180. doi: 10.1093/genetics/iyad180 (PMC10697816; doi:10.1093/genetics/iyad180)
Supplement: iyad180_Supplementary_Data [file iyad180_supplementary_data.zip › Movie_S1_Legend_GENETICS-2023-306511.docx]

**Supplementary Movie S1 Legend: Progeny of *unc-18(e81); uncp-18(syb6377)/+* animals.** Eggs laid by *unc-18(e81); uncp-18(syb6377)/+* animals were transferred from a plate onto the agar pad on a microscope slide with M9 butter, covered with a glass coverslip, sealed with Vaseline and imaged every 5 minutes for around 13 hours using a Zeiss Imager Z2 microscope with 63x objective. Note that the embryo on the top right does not pass through embryonic development as all other embryos do.
